# Quantitative sensory testing in a magnetic resonance environment: considerations for thermal sensitivity and patient safety

**DOI:** 10.3389/fpain.2023.1223239

**Published:** 2023-09-12

**Authors:** Ayeong (Jenny) Kim, Edina Szabo, Claire E. Lunde, Gabriela Comptdaer, David Zurakowski, Christine B. Sieberg, Scott A. Holmes

**Affiliations:** ^1^ Department of Anesthesiology, Critical Care, and Pain Medicine, Pain and Affective Neuroscience Center, Boston Children’s Hospital, Harvard Medical School, Boston, MA, United States; ^2^ Biobehavioral Pain Innovations Lab, Department of Psychiatry and Behavioral Sciences, Boston Children’s Hospital, Harvard Medical School, Boston, MA, United States; ^3^ Nuffield Department of Women’s & Reproductive Health, Oxford University, Oxford, United Kingdom; ^4^ Departments of Anesthesiology and Surgery, Boston Children’s Hospital, Harvard Medical School, Boston, MA, United States; ^5^ Department of Psychiatry, Harvard Medical School, Boston, MA, United States; ^6^ Pediatric Pain Pathway Lab, Department of Anesthesiology, Critical Care, and Pain Medicine, Boston Children’s Hospital, Harvard Medical School, Boston, MA, United States

**Keywords:** magnetic resonance imaging, thermal sensitivity, MR safety, quantitative sensory testing, pain

## Abstract

**Introduction:**

Quantitative sensory testing (QST) is often used to understand the perceptual basis of acute and chronic conditions, including pain. As the need grows for developing a mechanistic understanding of neurological pathways underlying perception in the basic and clinical sciences, there is a greater need to adapt techniques such as QST to the magnetic resonance (MR) environment. No studies have yet evaluated the impact of the MR environment on the perception of thermal stimuli. This study aimed to evaluate the differences in temperature sensitivity outside an MR environment and during an MRI scanning session. We hypothesized that there would be a difference in how participants reported their pain sensitivity between the two environments.

**Methods:**

Healthy participants underwent thermal QST outside the MR scanning environment, where they were asked to rate the temperature of a noxious stimulus at which they perceived their pain to be 7/10, using a Likert scale ranging from 0 to 10. Participants repeated this procedure inside a 3.0 T MRI approximately 30 min later. We repeated our investigation in a clinical cohort of participants with a chronic pain condition.

**Results:**

There were statistically significant changes of 1.1°C in thermal sensitivity between environments. This increase in pain threshold was found in healthy participants and replicated in the clinical cohort.

**Discussion:**

Findings can be applied toward improving MR safety, the resolution of brain pathways underlying pain mechanisms, and to more broadly comment on the impact of the MR environment on investigations that integrate perception-influenced processes.

## Introduction

The application of quantitative sensory testing (QST) in the magnetic resonance (MR) environment is the advancement of a well-studied technique into a novel environment that is promising for informing the mechanisms underlying cognitive processes. The MR environment is inherently complex, both in terms of the basic machinery and the impact it can have on the human body. As higher magnetic field environments are emerging worldwide (1), it is critical to understand the impact of the MR environment on cognitive faculties. Exposing patients to the MR environment means exposure to unique thermal (e.g., continuous flow of dehumidified room temperature air through the MR bore) and psychological environments (2, 3) and the established impact of MR and changing MR fields on the human body (4). There is little information on applying QST in the MR environment, particularly concerning complex cognitive processes such as pain perception.

Applying QST in pain cognitive neuroscience has proved pivotal in detecting clinical changes and informing pain mechanisms. Thermal QST is a valuable tool used in pain research and can help detect pathophysiological mechanisms associated with neuropathic pain (5) and detect altered central pain processing (6, 7). QST is safe and has been used in adult and pediatric cohorts (8), making it an increasingly popular method of testing pain sensitivity in patients among clinicians and researchers. The application of QST has shown to be reliable over time, including research showing the stability of the technique over a 10-week period (9). The predictive value of QST for identifying persons at risk of developing chronic pain has also been explored, with some research supporting the use of QST in predicting persistent post-surgical pain (10, 11). The application of QST has notable potential in focused populations and should be investigated for its’ application in unique environments, such as the MR environment.

To date, there have been no studies evaluating changes in thermal pain sensitivity using QST-based approaches in the MR environment. This study aimed to explore the relationship between thermal sensitivity inside and outside the MR environment using a standard QST pain sensitivity protocol that required participants to rate their level of perceived pain using a presented Likert scale. We conducted our investigation in the context of a group of healthy female participants between the ages of 13 and 43 years and extended our findings to a clinical cohort of participants with chronic pain. Our clinical cohort was comprised of patients with surgically confirmed endometriosis, a chronic pain condition that impacts approximately 1 in 10 women of reproductive age (12). Findings are discussed in terms of the application of QST in MR environments and the broader impact of MR on cognitive neuroimaging.

## Materials and methods

### Participants and study recruitment

Sixty-one participants were recruited for the study, and 55 (aged 13–43 years; *M* = 27.60, *SD *= 7.92) were included in the data analysis. Participants were required to be between the ages of 12–44 on the day of the study visit, with the ability to speak sufficient English to complete study visit tasks (i.e., self-report questionnaires in English). The clinical cohort included patients with surgically confirmed endometriosis, verified by research staff.

**Figure 1:**
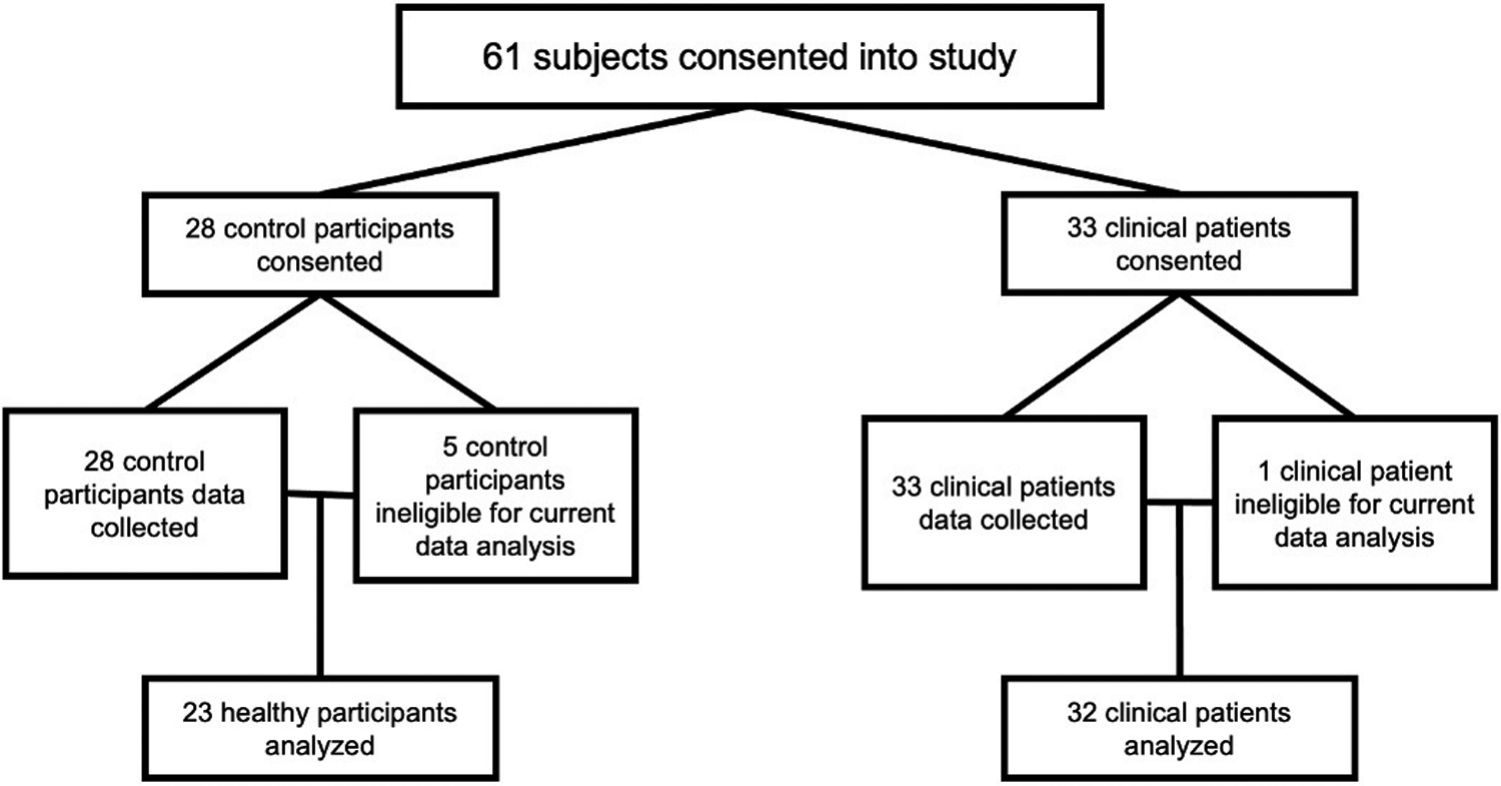
Participant analysis cohort breakdown. All participants are female sex assigned at birth and all clinical participants had surgically confirmed endometriosis.

Exclusion criteria were (1) inability to speak sufficient English to complete study visit tasks; (2) severe cognitive impairments; (3) patients with co-morbid medical and/or pain conditions that may potentially confound the data; (4) MRI exclusion criteria [e.g., metallic implants, claustrophobia, weight >350 lbs (limit of MRI table)]; (5) current use of opioid analgesics, which may confound pain data; (6) history of hysterectomy or oophorectomy; and (7) participants who are already in menopause, defined as the cessation of menses for 12 consecutive menses, unrelated to exogenous hormonal influences. Participants consented to the study after being screened for MRI compatibility and study eligibility by trained research staff. Six participants were excluded from the analysis due to withdrawal by self-request, positive drug screens, and equipment issues (see Figure 1).

Participants were female sex assigned at birth, most identified as female, with one clinical patient identifying as a transgender male and one as non-binary. Most participants identified as White (80%), with Asian being the second most prominent racial group (see Table 1). Participants were screened for birth control use and menstrual cycle history to minimize hormonal influences. All participants were either on hormonal birth control (i.e., combined birth control pill, hormonal intrauterine device (IUD), progesterone implant) or asked to come in between days 2 and 10 of their menstrual cycle, in which day 1 of the cycle was defined as the first day of their most recent period.

**Table 1:** Demographic breakdown of healthy and clinical participants.

Demographics of clinical vs. control subjects		
	Clinical	Control
	(*N* = 32)	(*N* = 23)
Age		
Minimum	15.68	13.03
Maximum	43.10	41.78
Mean	27.92	27.16
SD	8.43	7.33
Gender identity		
Female	93.8%	100.0%
Ethnicity		
Non-Hispanic	93.8%	73.9%
Hispanic	6.2%	26.1%
Race		
White	96.9%	56.5%
Asian	3.1%	21.7%
Black or African-American	6.3%	4.3%
Native American/Alaskan	6.3%	0.0%
Other	0.0%	17.4%

On the day of the study, participants were asked to report their pain levels from 0 (“no pain at all”) to 10 (“worst pain imaginable”). Healthy control participants reported pain scores of 0–2 (*M* = 0.18, *SD* = 0.59), while clinical participants reported average pain scores of 0–6 (*M *= 1.93, *SD *= 1.81). Participants were given a battery of psychological questionnaires, which included select PROMIS measures (anxiety and depression) (13, 14), the Pain Catastrophizing Scale (PCS) (15, 16), and the Fear of Pain Questionnaire (FPQ) (17, 18), with pediatric or adult versions administered accordingly.

### Psychological measures

#### Anxiety and depression measures

##### Anxiety

The PROMIS Anxiety 8a—Adult v1.0 (13) and Anxiety 8a—Pediatric v2.0 (14) were used to evaluate anxious symptomatology. This eight-item measure evaluates feelings of anxiety experienced within the last 7 days. Each question on the anxiety PROMIS measure includes a five-point Likert scale, ranging in value from one (Never) to five (Always). Raw scores are summed from responses, and then *T*-scaled to the general population. A *T*-score of 50 (*SD* = 10) represents the average score of the population. A higher PROMIS anxiety *T*-score represents more impaired functioning with a score of 60 or above being clinically elevated. Cronbach's alpha of the adult sample was 0.939 and for the pediatric sample was 0.953, demonstrating excellent internal item reliability in measuring anxiety and depression.

##### Depression

The PROMIS Depression 8a—Adult v1.0 (13) and Depression 8a—Pediatric v2.0 (14) were used to evaluate depressive symptomatology. Participants self-reported depressive feelings experienced within the last 7 days. This measure is similar in question format and scoring methodology as the PROMIS anxiety measure. A higher PROMIS depression *T*-score also represents more depressive symptoms with a *T*-score of 60 or above being clinically elevated. Cronbach's alpha for the adult sample was 0.958. Cronbach's alpha for the pediatric sample was 0.913.

#### Pain measures

##### Pain catastrophizing

The PCS (15) was used to measure pain catastrophizing, a construct assessing pain-related worry (19). It is scored on a Likert style scale from 0 (Not at all) to 4 (All the time) and is scored by summing the responses. Along with a total score, the PCS possesses three subscale scores assessing rumination, magnification, and helplessness. A total score of 30 and above represents clinically relevant catastrophizing. The Pain Catastrophizing Scale for Children (PCS-C) (16) is the pediatric version of the adult PCS. The PCS-C assesses the same symptomatology of pain catastrophizing and is well-validated in the literature for assessing pain-related rumination, magnification, and helplessness in a pediatric cohort. The Cronbach's alpha for the adult sample was 0.950. Cronbach's alpha for the pediatric sample was 0.921.

##### Fear of pain

The FPQ (17) measures self-reported feelings of fear of different noxious stimuli and is divided into three subscales of severe, minor, and medical pain. The questionnaire shows good internal and test–retest reliability. The Cronbach's alpha for the adult sample was 0.945. The Cronbach's alpha for the pediatric sample was 0.940. The Fear of Pain Questionnaire, child version (FOPQ-C) (18) was developed for pediatric patients and is also a five-point Likert scale (0 = “strongly disagree”; 4 = “strongly agree”) consisting of two subscales, fear of pain and avoidance of activities.

### Thermal QST

The noxious thermal stimulus was administered using an fMRI-compatible Medoc TSA 2 (Medoc, Israel), with a 30 mm × 30 mm thermode. The stimulus was applied on the medial and lateral sides of their non-dominant calf. Tested temperatures ranged from 32.0°C to 48.0°C, and the temperature rate of change was 13.0°C/s. Unless a task was being run, the thermode was kept at 32.0°C. The researcher would use a non-toxic pink highlighter to mark four spaces for thermal testing: two space medial and two spaces lateral on the non-dominant calf. The researcher would switch the space used during the threshold test for each trial in a circular, clockwise pattern from where the researcher started. The space (upper versus lower medial or lateral space) was pseudorandomized per participant.

Outside of the MRI environment, in a controlled testing space, participants were instructed to rate the pain from the stimulus using the pain scale of 0 (“no pain at all”) to 10 (“the worst pain imaginable”). The temperatures of both testing environments, inside and outside the MR environment, were thermostatically controlled to be approximately 21°C. Participants were given a remote-control device connected to the Medoc that allowed them to cease temperature changes when they felt a 7 out of 10 (moderate to severe pain) pain level from the noxious stimuli. Temperature paradigms were performed online and coincident with participants rating their level of pain perception. Three trials (Trial 1, Trial 2, and Trial 3) were performed to find the participants’ individual 7 out of 10 (7/10) temperature, changing locations of the thermode on the calf in between each trial. The average 7/10 temperature was found from averaging all three trials. A thermal training paradigm was performed after finding the 7/10 temperature in preparation for further testing inside the MRI testing. All trials and averaged 7/10 temperature outside the MRI are referred to as the outside temperature thresholds.

### MRI scanner protocol

**Figure 2:**
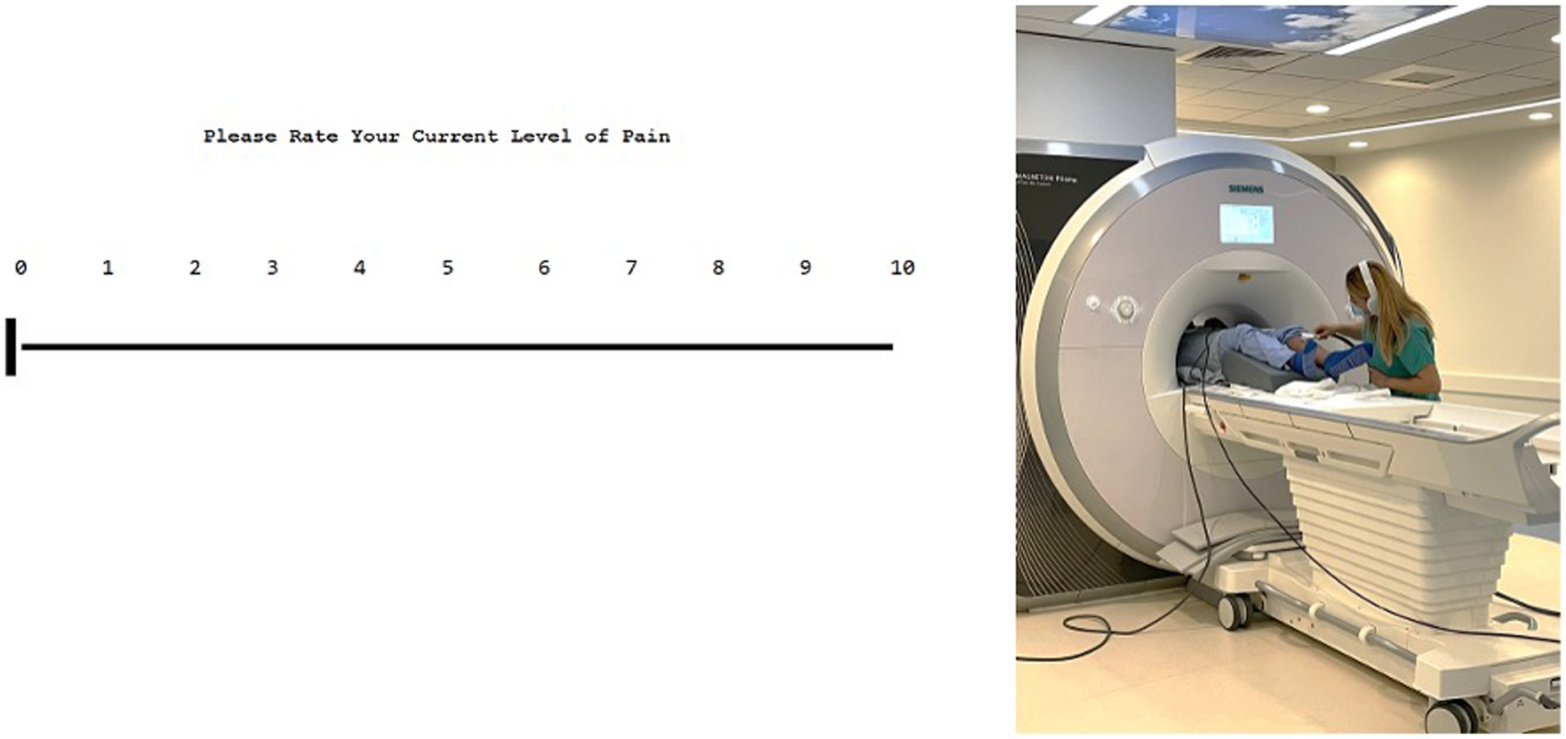
User interface of the eVAS (on the left) and example of inside MRI QST (on right). The interface is user-friendly and is controlled by subjects. Outside of the MRI, pain is rated via the eVAS using two keys that are marked with up and down arrows to move the slider on the scale down (less pain) and up (more pain). Inside the MRI, participants use the NNL grips to change this scale using two buttons, wherein one is to move the slider on the scale up and the other down. Participants are given instructions prior to beginning the task on proper use. This eVAS allows us to evaluate pain levels during the task. On the right, a research assistant is shown holding the thermode on the participant during the thermal QST protocol performed inside the MR environment.

Image acquisition was performed with a 3.0 T MRI (Siemens Prisma) scanner with a 64-chanel head coil. A 7 min and 02 s T1-weighted magnetization-prepared rapid acquisition gradient echo (MPRAGE), a 3 min and 07 s T2-weighted anatomical images, and a 7 min and 19 s resting-state sequence were performed (total time: 17 min and 28 s sequence). Participants were given similar verbal instruction and rated their pain with grips connected to a Nordic Neuro Lab (NNL) system that allowed participant control on an electronic visual analog scale (eVAS) from 0 to 10 (see Figure 2). When the participant reached a 7, a trained researcher stopped the thermode from changing temperature manually. The researcher holding the thermode inside the MRI briefly removed the thermode to change locations on the calf, as was performed outside. Skin was briefly wiped with a towel to remove perspiration in between each trial. Three trials were performed, and mathematical averaging was done to determine an average 7/10 temperature. These 7/10 temperatures are referred to as inside temperature thresholds. Participants were not administered blankets or sheets for the duration of the study to reduce excess overheating.

### Statistical analyses

Statistical analyses were performed using SPSS v. 24 (IBM, Armonk, NY, USA). Descriptive data were collected for demographics, emotional functioning, and thermal data. Shapiro–Wilk tests were used to examine normality. Fisher's exact test (chi-square test of homogeneity) was performed to examine racial breakdown proportions between cohorts. Normally distributed psychological data were compared with independent samples *t*-tests, while non-normally distributed data were compared via Mann–Whitney *U* tests. A related-samples Wilcoxon signed rank test was performed on the combined cohort data, as well as the separate clinical and control cohorts to evaluate whether there were differences between temperature threshold due to the MRI environment. We performed two-way ANOVA in order to explore an interaction effect between cohorts and MR environment. We calculated effect sizes using whole group data at 80% power and an alpha level of 0.05 to understand the effect of environment given our sample size.

## Results

Control participants were aged 13–42 years (*M* = 27.16, *SD *= 7.33), while clinical participants were aged 16–42 years (*M* = 27.92, *SD* = 8.43). All participants were female sex assigned at birth. There were significant differences (*p *< 0.01) based on race between the control and clinical cohorts. About 97% of the clinical cohort identified as White, and 0% identified as Other; 57% of control participants identified as White, and 17.4% identified as Other (see Table 1). Both groups had similar proportions of minority populations (i.e., Black/African-American, Asian).

Anxiety *T*-scores of control participants ranged from 37.10 to 74.90 (*M *= 51.88, *SD *= 9.83), while *T*-scores of the clinical cohort ranged from 37.10 to 73.00 (*M *= 58.97, *SD *= 7.93). Depression *T*-scores of control participants ranged from 37.10 to 72.60 (*M *= 45.68, *SD *= 8.77), while *T*-scores for the clinical cohort ranged from 43.20 to 73.60 (*M *= 55.93, *SD *= 8.04). Anxiety and fear of pain scores were normally distributed (*p *> 0.05), but depression and pain catastrophizing were not (*p *< 0.05). The clinical cohort endorsed greater symptomatology relative to healthy controls in anxiety, depression, and pain catastrophizing (*p *< 0.005). There were no significant differences between the groups in fear of pain. Anxiety, depression, and pain catastrophizing were statistically significantly different in between chronic pain and pain-free cohorts (*p* < 0.01), while fear of pain was not found to be statistically significant (*p* = 0.56) (see Table 2).

**Table 2:** Differences between clinical and control participants in psychological tests.

	Clinical			Control			Statistic	
Normal data	M	SD		M	SD		*t* (53)	*p*
Anxiety (*T*-score)	58.97	7.93		51.88	9.83		−2.957	0.005
Fear of pain	70.09	18.17		67.04	20.12		−0.587	0.56
Non-normal data	M	SD	Mean rank	M	SD	Mean rank	Test statistic	*p*
Depression (*T*-score)	55.93	8.04	17.46	45.68	8.77	35.58	4.149	<0.001
Pain catastrophizing scale	30.44	11.23	18.36	19.91	9.87	34.91	3.788	<0.001

The temperatures of both testing environments were thermostatically controlled to be approximately 21°C. A paired-samples Wilcoxon signed rank test was performed on the combined (healthy and clinical) cohort data to evaluate whether there were differences in temperature threshold due to the MRI environment. This was performed for each trial and an averaged trial. Statistically significant results (*p* < 0.005) were found in each trial and in the average of all three trials (see Table 3). As shown in Figure 1, for the combined cohort (healthy controls and clinical participants), there was a significant increase in temperature threshold inside of the MRI scanner for each trial and averaged data (trial 1: 1.5°C, *z *= 3.14; trial 2: 2.4°C, *z *= 4.32; trial 3: 0.4°C, *z *= 3.11; averaged: 1.1°C, *z *= 4.36).

**Table 3:** Related-samples Wilcoxon signed rank test to compare environments.

	Trial	*N*	Test statistic	*p*-value	Median	
					Outside MRI	Inside MRI
All participants	1	55	3.14	0.002	45.10	46.60
	2	55	4.32	<0.001	44.40	46.80
	3	55	3.11	0.002	45.50	45.90
	Averaged	55	4.36	<0.001	45.23	46.33
Clinical	1	32	2.28	0.023	45.75	46.50
	2	32	3.86	<0.001	44.60	46.90
	3	32	3.64	<0.001	45.80	46.45
	Averaged	32	4.03	<0.001	45.67	46.83
Control	1	23	2.05	0.040	45.00	46.90
	2	23	2.29	0.022	44.20	46.10
	3	23	0.76	0.445	44.20	44.90
	Averaged	23	1.98	0.048	43.70	46.23

Wilcoxon signed ranks tests were also performed within the clinical and control cohorts separately to determine if consistent differences inside and outside of the MRI were found in both cohort types. Statistically significant results were found in most trials (*p *< 0.05), except trial 3 of the control participants. Each trial indicated an increase [trial 1 (clinical, A): 0.75°C, trial 1 (control, B): 1.90°C; trial 2 (A): 2.30°C, trial 2 (B): 1.90°C; trial 3 (A): 0.65°C, trial 3 (B): 0.70°C; averaged (A): 1.16°C, averaged (B): 2.54°C] in the 7/10 threshold inside compared to outside of the MRI scanner (see Table 3).

To determine if there were differences in temperature thresholds between control and clinical participants, a Kruskal–Wallis *H* test was performed. There were no statistically significantly different 7/10 thresholds between cohort types inside of the MRI and outside of the MRI (*p* > .05) (see Table 4).

**Table 4:** Independent samples Kruskal–Wallis *H* test to compare independent cohorts.

	Trial	*N*	*df*	Test statistic	*p*-value	Median	
						Control	Clinical
Outside MRI	1	55	1	0.29	0.864	45.00	45.75
	2	55	1	0.21	0.621	44.20	44.60
	3	55	1	0.77	0.379	44.20	45.80
	Averaged	55	1	0.04	0.851	43.70	45.67
Inside MRI	1	55	1	0.25	0.619	46.90	46.50
	2	55	1	2.30	0.129	46.10	46.90
	3	55	1	2.90	0.088	44.90	46.45
	Averaged	55	1	1.53	0.216	46.23	46.83

### ANOVA

**Figure 3:**
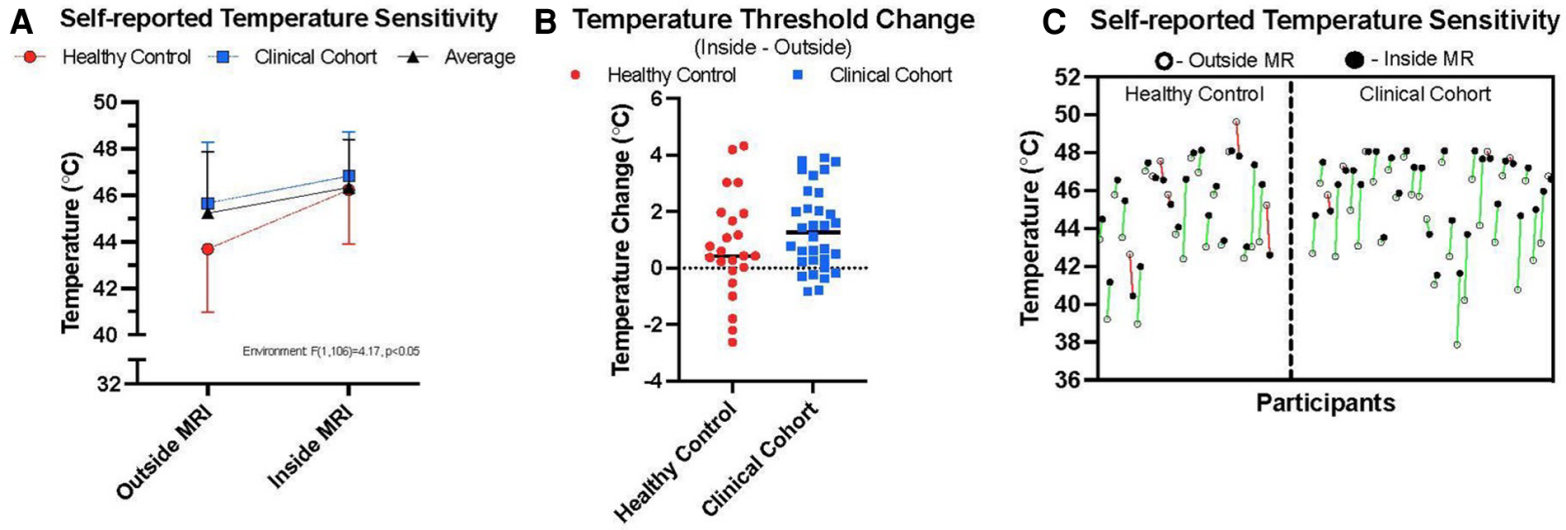
Thermal sensitivity. (**A**) outlines the average temperatures taken from median scores at which participants reported their 7/10 thermal sensitivity in the two environments, inside and outside of the MRI room. Healthy control data are presented alongside persons with chronic pain (clinical cohort) and averaged data across the two cohorts. (**B**) highlights the relative change between environments on an individual basis taking the difference between individual 7/10 thresholds between environments and a horizontal line plotting the median value for each cohort. (**C**) shows individual data points showing the relative change in thermal sensitivity ratings between the two environments for both cohorts (green = higher temperature sensitivities inside, relative to outside MR environment; red = lower temperature sensitivities inside, relative to outside the MR environment).

We elected to perform a two-way ANOVA to explore an interaction effect between cohorts and MR environment. There was no significant interaction between the environment and cohort for temperature threshold using averaged trial data (see Table 5 and Supplementary Table 1). There was no significant difference in temperature threshold between cohorts, *F*(1,106) = 0.732, *p *= 0.039, partial *η*^2^ = 0.007. The marginal means for control and clinical participants were 44.95 ± 0.36 and 45.35 ± 0.31, respectively, with a non-significant mean difference of −0.404 (95% CI, −1.34 to 0.53). There was a significant difference in environment, *F*(1,106) = 4.17, *p *< 0.05, partial *η*^2^ = 0.04, that being inside of the MRI was associated with a mean temperature threshold increase of 0.96 (95% CI, 0.28–1.90), with unweighted marginal means for outside and inside of the MRI being 44.67 ± 0.334 and 45.63 ± 0.33, respectively. Within the combined cohort, nine individuals were higher outside the MR environment, 44 inside the MR environment, and two reached the maximum temperature threshold in both environments (Figure 3).

**Table 5:** Results of a two-way ANOVA evaluating the interaction effects between environment and cohort on 7/10 threshold during an averaged trial.

Evaluating the interaction effects between environment and cohort on 7/10 temperature						
Averaged						
Predictor	Sum of squares	*df*	Mean square	*F*	*p*	Partial *η*^2^
(Intercept)	2,18,244.702	1	2,18,244.702	36,598.528	0	0.997
Environment	24.866	1	24.866	4.17	0.044	0.038
Cohort	4.365	1	4.365	0.732	0.394	0.007
Environment×Cohort	1.216	1	1.216	0.204	0.652	0.002
Error	632.1	106	5.963			

## Discussion

A reliable change in thermal pain sensitivity was experienced by participants based on the testing environment. The ability of QST to inform patient diagnostics and delineate central mechanisms of pain perception and chronicity is contingent upon the accurate application of thermal stimuli. To date, QST has largely been performed in clinical or research settings outside of the MR environment (20); however, the application of QST in the MR environment is an emerging application of the technology. The MR scanner represents an ideal environment for studying neurocognitive processes with unique psychological stressors (e.g., claustrophobia), novel stimuli, and attributes of the physics of the MR environment itself. The findings from this investigation outline a reliable change in thermal sensitivity that was experienced when both healthy and clinical participants completed a QST thermal sensitivity protocol inside and outside of the MR scanning environment.

The MR environment can present as stressful to participants, which can be exacerbated in people with underlying anxiety. Therefore, we explored two behavioral based interpretations of the findings. First, it is possible that the findings observed in the current investigation reflect active or exacerbated psychological symptoms. Participants in this investigation are actively screened for any history of claustrophobia and psychological co-morbidities that would require clinical intervention, as the condensed volume of the MR bore can induce feelings of anxiety (21) and other undesirable psychological symptoms (22, 23). In our investigation, our healthy control cohort reported relatively normal anxiety levels, with only three individuals reporting levels higher than mild. Similar findings were observed for clinical depression (see Table 2). Notably, our clinical cohort endorsed more symptoms of anxiety, depression, and pain catastrophizing compared to the healthy controls, demonstrating the impact of chronic pain on psychological wellbeing (24). This finding is aligned with previous literature, in which anxiety and depression are observed in populations with chronic pain (25–28). Moreover, previous literature has identified pain catastrophizing as a psychiatric measure associated with lower thermal tolerance (29), as well as increased pain sensitization (30). Despite these differences, we observed similar thermal sensitivity changes between cohorts (approximately 1.1°C; see Table 3). We also performed a correlation analysis between the psychological measures and the temperature thresholds inside and outside of the MRI to observe whether psychological backgrounds are associated with differences in temperature threshold. No statistically significant results were found (see Supplementary Table 6). However, we recommend that extra vigilance be taken with individuals who report elevated psychological symptoms to ensure patient comfort.

Second, observed findings may be due to practice-related effects, as all participants received outside thermal sensitivity testing prior to being tested in the MR environment. Contradicting this hypothesis are observations that practice effects (on the order applied in our investigation) may *increase* thermal sensitivity (31). That is, practice may *decrease* the temperature at which a person identifies a 7/10 pain threshold. Indeed, we saw *trends* in the data to suggest this was present on a trial-to-trial basis (decreased temperatures required to produce a 7/10) in this study (see Healthy Controls, Table 3); however, the change in environment was associated with a decrease in thermal sensitivity (higher temperatures required to produce a 7/10). Given that the MR environment is cooled, it would be expected that a person would require a lower temperature to induce a 7/10 as they accommodate to the environment—the reverse was observed. We note that a circulating, randomized, pattern of testing was performed to ensure that the exact same testing site was not used (see Materials and methods). As such, we suggest that the findings observed in this investigation likely do not pertain to the psychological nature of the MR scanning environment.

The MR environment has specific physics-based attributes that are known to impact the human body. As these have been known to produce thermal differences in human tissues, we explore their possible role in our findings. Radio-frequency pulses, used to produce the different imaging sequences, have established heating impacts (32, 33), leading to specific absorption rate (SAR) cut-offs established for scanning of human participants. SAR may cause heating in body tissues (34) as well as increases in skin temperature (35). These typically depend on the strength of the MR scanner (e.g., 3 vs. 7 T) and the length of the scanning sequence performed (36). As it pertains to this investigation, there was approximately a 30 min (anatomical scanning time + time of setup) delay between the testing of the two environments (see Supplementary MRI information). That is, between recording temperature sensitivity data outside of the scanner and reaching the point of the imaging paradigm inside the scanner, there were three different scanning sequences performed including an MPRAGE (7 min; 02 s), T2 (3 min; 07 s), and resting-state blood oxygenation level dependent (BOLD) echo planar imaging (EPI) (7 min; 19 s) scan. This exposure to different magnetic field environments could, in theory, produce warming of the human body. This would be notable in the context of changes in baseline operating of c-fiber pathways (37), the nerve fibers responsible for sending thermal nociceptive volleys throughout the nervous system to produce the perception of *pain*. Notably, prior work has shown that c-fiber pathways may act in response to changes in thermal stimuli, rather than to absolute temperature levels (31), suggesting that the local neuro-environment where recordings were made were unique in the two environmental conditions. As noted, we randomized the recording site between trials, so observations were most likely not the product of repeated administration of thermal stimuli from the MEDOC device in the same position, but the product of a more diffuse heating that modified the perception of the test stimulus across the multiple testing sites. We suggest that the impact of MR-based protocols on our participants may have impacted the mechanisms underlying pain perception.

An exploratory analysis was performed in which the main cohort was divided into a younger half (*n* = 27) and older half (*n* = 28). Most trials (2, 3, and averaged) showed statistically significant differences (*p* < 0.05) in 7/10 threshold temperature from inside and outside of the MRI scanner. The impact of the scanner environment was also explored after dividing the control and clinical cohorts into younger and older halves to identify age differences based on clinical cohort designation. No control cohorts showed statistically significant differences inside and outside of the MRI scanner when divided into younger and older halves (*p* > 0.05). However, clinical cohorts showed statistically significant differences inside and outside the MRI scanner for nearly all trials (except the older clinical cohort in trial 1). These follow-up analyses suggest that age did not impact current environment findings. At the overall cohort level, we continued to observe a 1°C difference between environments.

The presented findings have important implications for MR scanning in cognitive neuroscience. First, the issue of patient safety must be considered, particularly in the context of pediatric and special populations who cannot effectively communicate the presence or extent of pain. For these populations, there may be limited capacity to perform within-scanner thermal sensitivity testing based on abilities to understand instructions or behavioral limitations. These scenarios may present risk by applying thermal stimuli that is not proportionate to elicit an intended response. A significant risk factor for QST investigation is the possibility of thermal burns, which can be produced if the thermode is applied for too long, or at too high of a temperature. Given that findings from the current investigation reflect more thermal energy being needed to invoke the same level of pain stimulus as outside the scanner, there should be an abundance of caution as to how this metric is computed prior to application of noxious stimuli in the MR environment. Moreover, such stimuli need to be adjusted for application outside of the MRI if a person's 7/10 is only recorded inside of the MRI. Exposure to a within-scanner 7/10 is likely to exceed safety levels when performed outside of the MRI. Second, the use of QST for thermal sensitivity testing has important diagnostic and prognostic purposes and must be applied using strict criteria. Given that prior work on QST has noted standard deviations of patient reporting on the order of 1°–2° (38), we feel that a 1° change in thermal sensitivity is significant. With this degree of change, it is possible that a person's 7/10 threshold or their maximum pain intensity may be missed or over expressed in current and/or future protocols. This has translational effects for subsequent analyses on the validity and reliability of brain function and structure observations that demand application of procedures validated outside of the scanner. Future research should integrate findings from this investigation either to introduce a correction factor or perform similar test–retest data to understand the impact of MR environment on their study findings. Third, and most broadly, observed changes in thermal sensitivity reflect a change in pain perception to the applied nociceptive stimulus (temperature). If the observed changes are a direct result of the scanning environment, the implications of findings extend beyond the pain sciences. That is, research into specific faculties such as executive function and memory are not independent of perception and should consider, alongside the findings of their own investigations, the physical impacts of the MR environment. This is especially notable given the desire to increase resolution with higher field MR environments.

The current investigation has important limitations. First, the study population was solely represented by participants who were biologically women at birth. Although we have no reason to suggest the presence of this finding would be absent in men, the strength of its effect or characteristics may differ. Second, our study cohort was of moderate size. To our knowledge, we are the first to investigate the impact of scanner environment on temperature sensitivity, so we were unable to calculate proposed effect sizes. However, we demonstrated our findings in two independent cohorts (overall group effect size of 0.47—see Material and methods), one of which presented with a chronic condition that included elevated levels of anxiety and depression. As shown in prior research (39), there is significant inter-subject variability in QST ratings. This should be kept in mind when interpreting study findings considering our smaller cohorts; however, having intra-subject data between the two environments will help increase reliability in findings. Our clinical cohort was disproportionally represented by persons identifying as White, which reflects a current disparity in access to medical care for minority populations (40). Finally, this investigation was performed under specific scanner sequence protocols using a 3 T Siemens MR scanner. If study findings were due to the physical environment of the scanner, then any changes to these, perhaps by including a higher SAR scan (e.g., diffusion-weighted vs. MPRAGE), may modify study findings. It will be of high interest to evaluate this process in future research and ultimately control or correct for its impact on study findings.

In conclusion, this investigation found a significant and reliable change in thermal sensitivity based on the MR scanning environment. We point out these findings to be significant as they pertain to patient safety, the evaluation and diagnosis of pain patients, and the evaluation of broader cognitive neuroscience research questions that are impacted by a patient's perception in the MR environment. Our answers to questions involving basic science and disease mechanisms are dependent on the proper application of quantitative sensory testing in the MR environment.

## Data Availability

The raw data supporting the conclusions of this article will be made available by the authors, without undue reservation.
