# Supplementary material for: Quantitative sensory testing in a magnetic resonance environment: considerations for thermal sensitivity and patient safety
Source: Front Pain Res (Lausanne). 2023 Sep 12;4:1223239. doi: 10.3389/fpain.2023.1223239 (PMC10520956; doi:10.3389/fpain.2023.1223239)
Supplement: Supplementary file 1 [file Table1.docx]

**Correlation matrix examining correlations between 7/10 temperatures in both environments and psychological measures**

We elected to explore the correlation between changes in average temperature inside and outside of the MRI and our psychological measures to examine whether the changes observed may be due to the underlying psychological differences at the subject level, or the MRI environment itself. No statistically significant differences were observed between the psychological measures and the average 7/10 designated temperatures inside and outside of the MRI. Therefore, we believe that this further supports our hypothesis that the difference in temperature is induced by the MRI machine environment, and not from intrinsic behavioral profiles, as measured by our psychological battery.

**
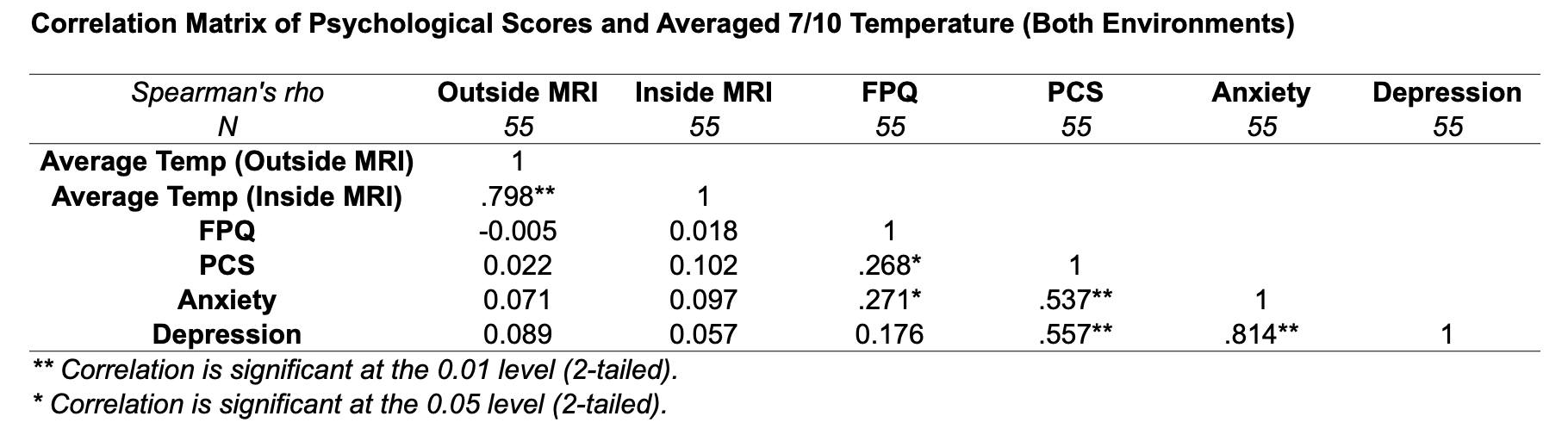
**

**Supplementary Table 1. Correlation Matrix of Psychological Scores and Averaged 7/10 Temperatures within Both Environments**
