# Supplementary material for: Quantitative sensory testing in a magnetic resonance environment: considerations for thermal sensitivity and patient safety
Source: Front Pain Res (Lausanne). 2023 Sep 12;4:1223239. doi: 10.3389/fpain.2023.1223239 (PMC10520956; doi:10.3389/fpain.2023.1223239)
Supplement: Supplementary file 2 [file Table2.docx]

**Expanded ANOVA statistics**

**
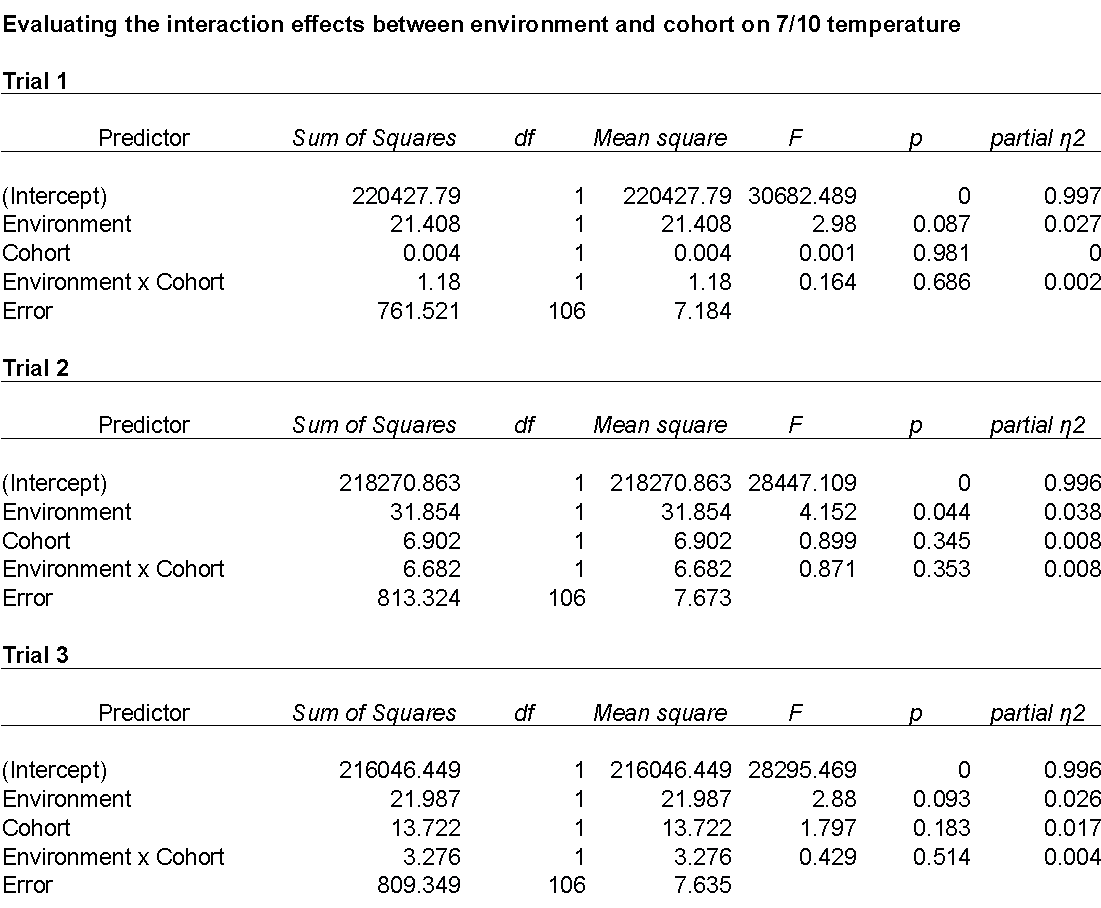
**

**Supplementary Table 2. ANOVA results per trial.**

Data are mean ± standard error unless otherwise stated. As determined earlier via Shapiro-Wilk's, the data were not normally distributed, but tests were performed with this knowledge. Levene's test for equality showed that variances were homogeneous (*p* > 0.05) in each trial and the averaged trial. There were no statistically significant interaction effects between environment and cohort for each trial (*p* > 0.05), nor the averaged trial (see Table 8). Therefore, the main effect of environment and cohort type were analyzed. All pairwise comparisons were run and were reported 95% confidence intervals, and *p*-values are Bonferroni-adjusted. Post hoc tests are not performed due to fewer than three groups.

In trial 1, there was no statistically significant difference in temperature inside or outside of the MRI scanner, *F*(1,106) = 2.980, *p* = .087, partial *η^2^* = .027. The unweighted marginal means of temperature for outside and inside the MRI were 44.93 ± .366 and 45.82 ± .366, respectively, with a non-statistically significant mean difference of -.894 (95% CI, -1.922 to .133). There was no statistically significant difference in temperature for pain-free controls and patients with clinical, *F*(1,106) = .001, *p* = .981, partial *η^2^*= .000. The marginal means for pain-free participants and participants with clinical were 45.383 ± .395, and 45.370 ± .335, respectively, with a non-statistically significant mean difference of .012 (95% CI, -1.015 to 1.039).

In trial 2, there was a statistically significant difference in temperature with regards to the environment, *F*(1,106) = 4.152, *p* < .05, partial *η^2^* = .038. Being inside of the MRI was associated with a mean temperature increase of 1.091 (95% CI, 0.29 to 2.153), with unweighted marginal means of temperature for outside and inside of the MRI were 44.608 ± .379 and 45.699 ± .379, respectively.

There was no statistically significant difference in temperature with the cohort, *F*(1, 106) = .899, *p* = .345, partial *η^2^*= .008. The marginal means for pain-free participants and participants with clinical were 44.90 ± .408 and 44.41 ± .346, respectively, with a non-statistically significant mean difference of -.508 (95% CI, -1.569 to .554).

In trial 3, there was no statistically significant difference in temperature inside or outside of the MRI scanner, *F*(1,106) = 2.880, *p* = .093, partial *η^2^* = .026. The unweighted marginal means of temperature for outside and inside the MRI were 44.47 ± .378 and 45.38 ± .378, respectively, with a non-statistically significant mean difference of -.906 (95% CI, -1.965 to .153). There was no statistically significant difference in temperature for pain-free controls and patients with clinical, *F*(1,106) = 1.797, *p* = .183, partial *η^2^* = .017. The marginal means for pain-free participants and participants with clinical were 44.565 ± .407, and 45.281 ± .345, respectively, with a non-statistically significant mean difference of -.716 (95% CI, -1.775 to .343).
